# Study on the Optimization of Hyperspectral Characteristic Bands Combined with Monitoring and Visualization of Pepper Leaf SPAD Value

**DOI:** 10.3390/s22010183

**Published:** 2021-12-28

**Authors:** Ziran Yuan, Yin Ye, Lifei Wei, Xin Yang, Can Huang

**Affiliations:** 1 Soil and Fertilizer Research Institute, Anhui Academy of Agricultural Sciences, Hefei 230031, China; yuanziran11@163.com (Z.Y.); yangxin20210928@163.com (X.Y.); 2 Key Laboratory of Nutrient Cycling and Resources Environment of Anhui Province, Anhui Academy of Agricultural Sciences, Hefei 230031, China; 3 Faculty of Resources and Environmental Science, Hubei University, Wuhan 430062, China; weilifei2508@hubu.edu.cn (L.W.); 201711110811067@stu.hubu.edu.cn (C.H.)

**Keywords:** pepper leaf, SPAD value, hyperspectral inversion, characteristic waveband selection

## Abstract

Chlorophyll content is an important indicator of plant photosynthesis, which directly affects the growth and yield of crops. Using hyperspectral imaging technology to quickly and non-destructively estimate the soil plant analysis development (SPAD) value of pepper leaf and its distribution inversion is of great significance for agricultural monitoring and precise fertilization during pepper growth. In this study, 150 samples of pepper leaves with different leaf positions were selected, and the hyperspectral image data and SPAD value were collected for the sampled leaves. The correlation coefficient, stability competitive adaptive reweighted sampling (sCARS), and iteratively retaining informative variables (IRIV) methods were used to screen characteristic bands. These were combined with partial least-squares regression (PLSR), extreme gradient boosting (XGBoost), random forest regression (RFR), and gradient boosting decision tree (GBDT) to build regression models. The developed model was then used to build the inversion map of pepper leaf chlorophyll distribution. The research results show that: (1) The IRIV-XGBoost model demonstrates the most comprehensive performance in the modeling and inversion stages, and its $R_{cv}^{2}$, $RMSE_{cv}$, and $MAE_{cv}$ are 0.81, 2.76, and 2.30, respectively; (2) The IRIV-XGBoost model was used to calculate the SPAD value of each pixel of pepper leaves, and to subsequently invert the chlorophyll distribution map of pepper leaves at different leaf positions, which can provide support for the intuitive monitoring of crop growth and lay the foundation for the development of hyperspectral field dynamic monitoring sensors.

## 1. Introduction

Chlorophyll content is one of the most important indicators of the health status of crops and is significant for guiding crop fertilization and field management in different crop growth periods [1]. SPAD values can be directly used as relative values to characterize chlorophyll content. A portable chlorophyll meter is usually used to measure the SPAD value of plant leaves to directly characterize the relative plant chlorophyll content. However, leaves need to be repeatedly inserted during the process, which makes large-scale chlorophyll detection using this method difficult. Research shows that the SPAD value can be used to accurately derive hyperspectral remote sensing data in a non-destructive and pollution-free manner at a low price. In recent years, hyperspectral remote sensing has become a powerful tool for chlorophyll content estimation. Because it is rapid, non-destructive, and capable of detecting chlorophyll over large areas, it is of great significance for crop growth monitoring, precise fertilization, and yield evaluation [2,3].

Hyperspectral imaging technology combines the advantages of both spectrum and image. It has a high resolution and multi-band capabilities. Further, it integrates an atlas, combining traditional imaging and spectral technologies [4]. Changes in plant chlorophyll content lead to changes in the plant reflectance spectrum characteristics [5]. The use of hyperspectral technology to obtain plant growth parameters provides a theoretical basis for measuring chlorophyll, which makes it possible to monitor the growth of crops across a large area [6]. Traditional chlorophyll determination methods mainly rely on chemical experiments, are labor intensive, consume a lot of material resources, and require sample destruction. Although portable chlorophyll meters can measure chlorophyll content in real time, they require manual and repeated measurements, which limits their application in the monitoring of large areas. Furthermore, portable devices can only provide information about the chlorophyll content at a certain point of the leaf, which is not sufficient to obtain an accurate whole-leaf chlorophyll concentration [7].

Hyperspectral technology can not only quantitatively predict the chlorophyll content of the plant leaf but also perform inversion research and image presentation on the distribution of the leaf’s chlorophyll content. Zhao et al. used this technology in combination with vegetation index analysis to develop a method that uses hyperspectral imaging technology to obtain five different images in real time to facilitate measurements of leaf water status, relative water content, and equivalent water thickness in tomato varieties [8]. Daughtry and Wu et al. analyzed the accuracy of more than 10 spectral indices, such as MCARI and OSAVI, to estimate the chlorophyll concentration in maize leaves [9,10]. Yu et al. collected samples of leaves, roots, and stems of pepper plants and determined the nitrogen content using a random frog algorithm combined with the partial least-squares method to establish the nitrogen content growth model of the pepper plant [11]. Their results show that hyperspectral imaging is a very promising technology and has great potential for determining the spatial distribution of nitrogen content in pepper plants.

However, there are no studies that use hyperspectral imaging to examine the differences in spatial distribution of SPAD values in leaves located at different positions on pepper plants. Therefore, to ascertain the response of pepper plants’ leaf chlorophyll spatial distribution during the growth process, this study adopted hyperspectral imaging technology to develop a method for diagnosing the SPAD value and mapping the spatial distribution of chlorophyll in leaves located at different positions. Four algorithms were used to screen the sensitive wavelengths of pepper leaf chlorophyll diagnosis. These were combined with four regression models to establish a SPAD value diagnostic model. This lays a foundation for the dynamic response of chlorophyll during the growth season of pepper plants.

## 2. Materials and Methods

### 2.1. Sample Collection

**Figure 1:**
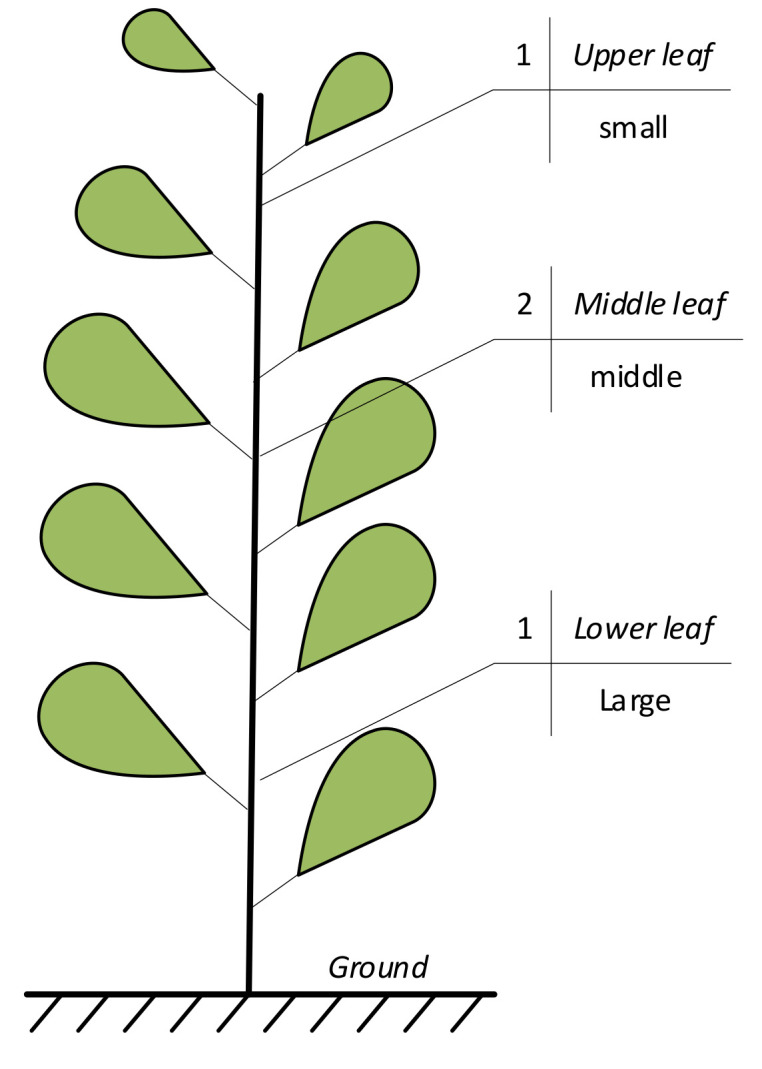
Pepper plant leaf position.

The study area was located in Wuhu Dehong Ecological Agriculture Co., Ltd. (118°12′ E, 31°26′ N), Shuangba Village, Shenxiang Town, Jiujiang District, Wuhu City, China. It has a subtropical temperate monsoon climate, with sufficient sunlight and rainfall. The experimental variety was Wanjiao 177, the planting time was 20 July 2020, and the collection time was 7 September 2020. The pepper samples were collected at the seedling stage. The fertilization level was selected according to the local conventional fertilization level. The pepper leaves are arranged in descending order according to the leaf growth sequence and are divided into upper, middle, and lower leaves. The upper leaves were the smallest in size, while the lower leaves were the largest. The size of the middle leaves was in between the sizes of the upper and lower leaves (Figure 1). The leaves of the pepper plants were artificially plucked. Fifty leaves were randomly collected from three leaf positions of different pepper plants. Hence, 150 leaf samples in total were placed in a sealed bag to keep the leaves fresh, and taken back to the laboratory immediately to obtain hyperspectral image data.

### 2.2. Chlorophyll Determination

**Figure 2:**
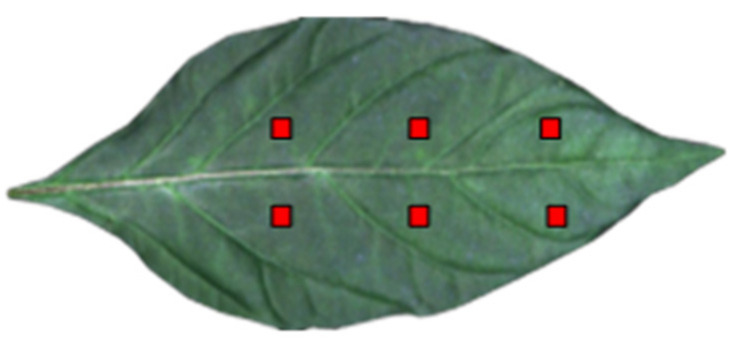
Sampling area of pepper leaves.

The SPAD-502 Plus chlorophyll meter (Konica Minoita, Tokyo, Japan) was used to measure chlorophyll content. SPAD values can be directly used as relative values to characterize chlorophyll content [12,13,14]. The chlorophyll meter has the following characteristics: measurement area: 2 × 3 mm^2^; measurement accuracy: ±1.0 SPAD unit; and measurement range: −9.9–199.9 SPAD unit. Each leaf was divided into six plots (as shown in Figure 2). Three measurements were recorded for each plot, and the average value was taken as the final result of the chlorophyll content of the leaves.

The formula used to calculate the SPAD values is shown in Equation (1):

(1) $$\mathrm{SPAD}=K\cdot \mathrm{lg}\left(\frac{IRt/IR0}{Rt/R0}\right)$$

where K is a constant; IRt is the incident 940 nm infrared light intensity passing through the blade; IR0 is the emitted infrared light intensity; Rt is the incident 650 nm red light intensity passing through the blade; and R0 is the emitted red light intensity.

### 2.3. Hyperspectral Data Collection

**Figure 3:**
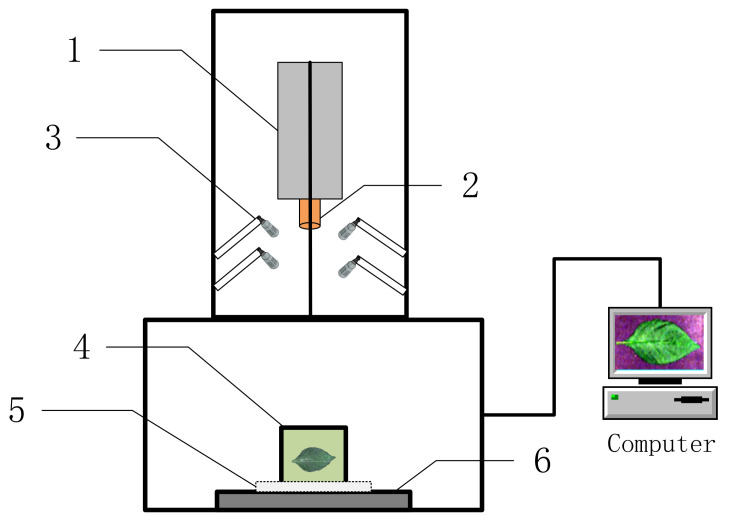
Schematic diagram of the GaiaSorter hyperspectral imaging system. 1. Hyperspectral imager, 2. imaging lens, 3. halogen lamp, 4. sample table, 5. correction whiteboard, and 6. electric translation table.

After completing the chlorophyll measurements, the leaves were cleaned with ultrapure water, and the excess surface water was removed using an absorbent paper. Figure 3 shows a schematic diagram of the hyperspectral imaging system used in this study (Wuxi Dualix Spectral Image Technology Co., Ltd. (formerly Sichuan Dualix Spectral Image Technology Co., Ltd.), Wuxi, China, Model: GaiaSorter). The imaging system mainly includes a tungsten halogen lamp as the light source, a hyperspectral camera, an electronically controlled mobile platform, a server and computer control, and other parts.

The height between the hyperspectral camera and the displacement platform was 60 cm, and the height between the halogen tungsten light source and the displacement platform was 40 cm. The wavelength range was 400–1000 nm, and the spectral resolution was 3.6 nm. Experiments were performed in a dark box to perform image correction on the collected spectral images. The image correction formula is given in Equation (2).

(2) $$R_{ref}=\frac{DN_{raw}-DN_{dark}}{DN_{white}-DN_{dark}}$$

where $R_{ref}$ is the corrected image, $DN_{raw}$ is the original image, $DN_{white}$ is the whiteboard image, and $DN_{dark}$ is the blackboard corrected image.

### 2.4. Spectral Extraction

**Figure 4:**
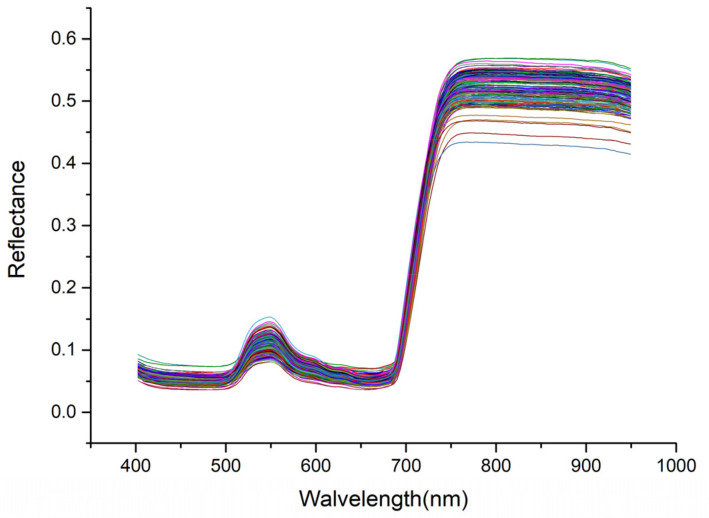
Original spectral curve.

ENVI 5.3 was used to read the hyperspectral image data of pepper leaves and select six representative rectangular regions of interest (avoiding leaf veins) in the image (Figure 2) as the original spectrum of the sample. A weighted average spectrum was also obtained, which was used as the original spectral data (Figure 4).

### 2.5. Research Methods

#### 2.5.1. Correlation Coefficient Method

Spearman’s correlation coefficient, which is an index that measures the association between two sets of variables, was used to describe the relationship between the spectral characteristics and SPAD value of pepper leaves [15,16]. We used a monotonic equation to evaluate the correlation between the two statistical variables. The formula used is shown in Equation (3):

(3) $$\rho =\frac{\sum_{i=1}^{N}\left(x_{i}-\bar{x})(y_{i}-\bar{y}\right)}{\sqrt{\sum_{i=1}^{N}{\left(x_{i}-\bar{x}\right)}^{2}}\sum_{i=1}^{N}{\left(y_{i}-\bar{y}\right)}^{2}}$$

where ρ represents the correlation coefficient, $x_{i}$ is the reflectance of the ith band, $y_{i}$ is the SPAD value of the ith leaf sample, $\bar{x}$ is the average reflectance, and $\bar{y}$ is the average SPAD value of the pepper leaves.

#### 2.5.2. Stability Competitive Adaptive Reweighted Sampling (sCARS)

sCARS is an advanced wavelength selection method that gradually removes unimportant variable information to achieve the purpose of collecting informative variables [17,18]. The algorithm defines the critical wavelength as the wavelength with the largest absolute value of the regression coefficient in a multivariate linear model (such as PLSR). sCARS can be summarized as follows:

1. Select N wavelength subsets from N Monte Carlo sampling [19] runs in an iterative and competitive manner. In each sampling process, a fixed proportion of samples is randomly selected to establish a calibration model.
2. Perform a two-step process to select characteristic wavelengths: use an exponential decrease function [17] for wavelength selection and use adaptive reweighted sampling to achieve competitive wavelength selection.
3. Use cross-validation [20] to select the subset with the smallest cross-validation root mean square error (RMSECV).

#### 2.5.3. Iteratively Retaining Informative Variables

Iteratively retaining informative variables (IRIV) is a feature variable selection algorithm based on the binary matrix shift filter (BMSF) [21]. Each row of the matrix (containing random combination of the variables) separately establishes partial least-squares models and uses RMSECV to evaluate the effect of different random variable combination models [22,23]. Based on the model cluster analysis method, the average value of RMSECV with and without the variable is calculated for each wavelength, and the difference between the two, known as the difference of mean values (DMEAN), is obtained. The non-parametric test method, Mann–Whitney U test, is used for hypothesis testing [22,24]. Each iteration generates different DMEAN and p values. Both the strongly and weakly informative wavelength variables are retained. After multiple iterations, the uninformative wavelength variables and interfering wavelength variables are eliminated, and finally, reverse elimination is performed to obtain the optimal characteristic wavelength variable.

Step 1: The raw data of m samples of p variables are formed into a matrix A containing only the numbers 0 and 1, where the number 1 represents a variable used for modeling, and the number 0 means that the variable was not used for the modeling. The RMSECV value obtained by five-fold cross-validation was used as the evaluation standard, and the vector of m×1 size was recorded as $RMSECV_{0}$. substitute 1 in the *i*th column (*i* = 1, 2, ..., p) of matrix A for 0, and 0 for 1 to obtain matrix B. The partial least squares (PLS) model is also established in each row of matrix B, and the vector of m×1 size is recorded as $RMSECV_{i}$.

Step 2: Define $\varphi_{0}$ and $\varphi_{i}$ to evaluate the importance of each variable as follows:

(4) $$\varphi_{0k}=\left\{\begin{array}{c} k^{th}RMSECV_{0}\\ k^{th}RMSECV_{i} \end{array}\right.\begin{array}{c} A_{ki}=1\\ B_{ki}=1 \end{array};\varphi_{ik}=\left\{\begin{array}{c} k^{th}RMSECV_{0}\\ k^{th}RMSECV_{i} \end{array}\begin{array}{c} A_{ki}=0\\ B_{ki}=0 \end{array}\right.$$

where $k^{th}$ represents the *k*th line in the vector, and the $k^{th}RMSECV_{0}$ and $k^{th}RMSECV_{i}$ represent the values of the *k*th row in the vectors $RMSECV_{0}$ and $RMSECV_{i}$, respectively. The mean values of $\varphi_{0}$ and $\varphi_{i}$ are denoted as $M_{i,in}$ and $M_{i,out}$, respectively, and the two mean values are subtracted to obtain $DMEAN_{i}$. If $DMEAN_{i}<0$, it is a strongly informative variable or a weakly informative variable; if $DMEAN_{i}>0$, it is an uninformative variable or an interfering variable.

(5) $$DMEAN_{i}=M_{i,in}-M_{i,out}$$

p = 0.05 was defined as the threshold for the Mann–Whitney U test [21], where the p value, denoted as $p_{i}$, is computed by the Mann–Whitney U test with the distribution of $\varphi_{0}$ and $\varphi_{i}$. The smaller the $p_{i}$ value, the more significant the difference between the two distributions. Finally, the variables were divided into the four categories (strongly informative variables, weakly informative variables, uninformative variables, and interfering variables).

Step 3: Strongly informative variables and weakly informative variables are retained for each iteration, and uninformative variables and interfering variables are eliminated, so that a new subset of variables is generated. Return to step 1 for the next iteration until there are only strong and weak informative variables left. The defined variable types are listed in Table 1.

Step 4: The backward elimination of the reserved variables is undertaken as follows:

(a) Denote t as the number of reserved variables.

(b) For all the reserved variables, obtain the RMSECV value with five-fold cross-validation using PLS, which is denoted as $\theta^{t}$.

(c) Leave out the ith variable and apply five-fold cross-validation to the remaining t−1 variables to obtain the RMSECV value $\theta_{-i}$. Conduct this for all variables i=1,2,…,t.

(d) If $\min\{\theta_{-i},1\leq i\leq t\}>\theta^{t}$, step (g) is performed.

(e) When excluding the ith variable with the minimum RMSECV value, remove the ith variable and change t to be t−1.

(f) Repeat steps (a)–(e).

(g) The remaining variables are the final informative variables.

#### 2.5.4. Partial Least-Squares Regression

Partial least-squares regression (PLSR) is a spectral analysis method that includes multiple linear regression, canonical correlation analysis, and principal factor analysis. The main objective of PLSR is to establish a linear model of independent variables, particularly in cases where two groups containing a large number of highly linearly correlated variables are analyzed. PLSR is also used when the number of samples is less than the number of variables to avoid overfitting [25,26,27]. The principle of PLSR is as follows. First, extract the mutually independent components $\left(x_{1},x_{2,\ldots ,}x_{m}\right)$ from the independent variable $T_{h}(h=1,2,\ldots )$. The extracted principal components carry as many original components as possible. Then, extract the independent components $\left(y_{1,}y_{2},\ldots ,y_{m}\right)$ from the independent variable $U_{h}=(h=1,2,\ldots )$. The covariance between $T_{h}$ and $U_{h}$ must be maximized, and the regression equation between the extracted components and the dependent variable is established through the multiple regression method. The basic model of the PLSR is:

(6) $$X=T_{h}P^{T}+E$$

(7) $$Y=U_{h}Q^{T}+F$$

where P and Q are m×h orthogonal load matrices, and E and F are error terms, which are random variables that follow a normal distribution.

#### 2.5.5. Extreme Gradient Boosting (XGBoost) 

XGBoost is a distributed gradient boosting algorithm based on classification and regression trees. XGBoost is popular in the fields of machine learning and data mining and has excellent judgment and recognition capabilities. The basic principle is to weigh the results of multiple decision trees (weak classifiers) as the final output (strong classifier) [28]. XGBoost achieves good control of model complexity by adding rule items to the objective function, thereby solving the problem of collinearity between the variables to a certain extent and preventing overfitting of the model. In the XGBoost model, the second-order Taylor series is used for the cost function, and the first-order and second-order derivatives are used to approximate the optimization of the objective function closer to the actual value, thereby improving the prediction accuracy [29,30].

#### 2.5.6. Random Forest Regression (RFR)

RFR is an integrated statistical learning classification and regression algorithm that combines multiple decision trees to produce similar predictions for different features of the same phenomenon [31]. The output is the average of all the decision tree results in a random forest, assuming that the training set is independently extracted from the distribution of random vectors. The prediction result of the model is the mean of the k regression trees.

#### 2.5.7. Gradient Boosting Decision Tree (GBDT)

GBDT is a comprehensive algorithm with a strong learning strategy. Although the original purpose was to solve the classification problem, it has been successfully applied in the field of regression [32,33].

(8) $$F_{m}(x)=F_{m-1}(x)+h_{m}(x)$$

Here, $h_{m}(x)$ represents the basic functions of the weak learners. In GBDT, the basic function $h_{m}$ is a small regression tree of fixed size, and the GBDT model $F_{m}(x)$ can be regarded as the sum of m small regression trees. A new tree is generated for each iteration, m. A simple tree is determined by the deviation between the experimental measurements and all previous model (i.e., gradient) predictions. Then, the regression tree is incorporated into the GBDT model.2.5.8. Software

CA, sCARS, and IRIV were programmed in MATLAB Version 2017b. SPXY and the regression models (PLSR, XGBoost, RFR, GBDT) were written in Python/Jupyter Notebook. The machine learning algorithms in the scikit-learn packages were also used.

### 2.6. Accuracy Evaluation

A 10-fold cross-validation was used to evaluate the accuracy of the model. The original dataset was randomly divided into 10 subsets with approximately equal sample sizes. Nine of them were combined as the training set in turn, and the one remaining set was used as the test set. In each test, the evaluation index, such as the correct rate, was calculated, and the generalization ability of the model was evaluated by taking the average value of the evaluation index after k tests. The parameters of determination coefficients ($R_{cv}^{2}$), root mean square error ($RMSE_{cv}$), and mean absolute error ($MAE_{cv}$) generated by 10- fold cross validation were used to measure the accuracy of the models. The closer $R_{cv}^{2}$ is to 1, the better the stability of the model and the higher the degree of fit. The $RMSE_{cv}$ and $MAE_{cv}$ were used to test the predictive ability of the model. The smaller the $RMSE_{cv}$ and $MAE_{cv}$, the better the predictive ability.

(9) $$R^{2}=\left(\frac{\sum_{i=1}^{n}\left(x_{i}-\bar{x})(y_{i}-\bar{y}\right)}{\sqrt{\sum_{i=1}^{n}{\left(x_{i}-\bar{x}\right)}^{2}}\sqrt{\sum_{i=1}^{n}{\left(y_{i}-\bar{y}\right)}^{2}}}\right)$$

(10) $$RMSE=\sqrt{\frac{\sum_{i-1}^{n}{\left(y_{i}-\hat{y_{i}}\right)}^{2}}{n}}$$

(11) $$MAE=\frac{1}{m}\sum_{i=1}^{n}\left|y_{i}-\hat{y_{i}}\right|$$

where n is the number of samples, $y_{i}$ is the measured value, $\hat{y_{i}}$ is the predicted value, and $\bar{y}$ is the average of the measured values.

### 2.7. Technical Roadmap

**Figure 5:**
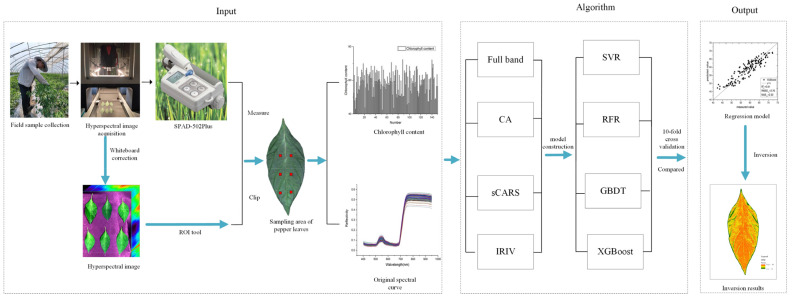
Technical Roadmap.

In this study, 150 samples of pepper leaves with different leaf positions were selected as the research object, and the hyperspectral image data and chlorophyll content of the pepper leaves were obtained. The technical roadmap is illustrated in Figure 5. The hyperspectral images were first white-calibrated, and then the original spectral data were obtained through the region of interest. The CA, sCARS, and IRIV methods were used, respectively. The IRIV screens the characteristic bands and uses PLSR, XGBoost, RFR, and GBDT to construct regression models. A 10-fold cross-validation was used as the accuracy evaluation index to filter out uninformative variables. The optimal algorithm reuses the constructed model to establish the inversion map of pepper leaf chlorophyll distribution, which lays the foundation for exploring the dynamic response of pepper chlorophyll during the growth period.

## 3. Results

### 3.1. Selection of Characteristic Band Based on CA Algorithm

**Figure 6:**
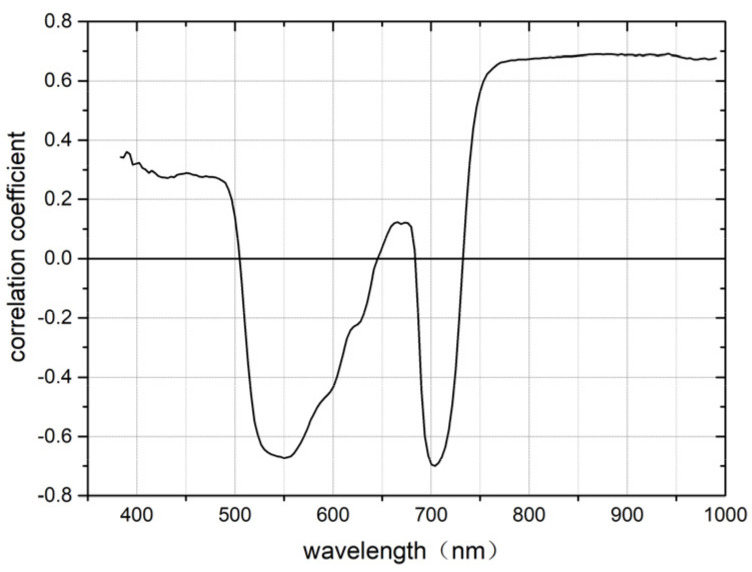
Correlation of SPAD values and spectral reflectance.

Spearman’s correlation analysis was performed between the original spectral reflectance of the whole wave band (400–1000 nm) and the SPAD values of pepper leaves. The spectral reflectance of each band was correlated with the SPAD value and a correlation curve was drawn. As shown in Figure 6, the overall correlation was relatively high, and the volatility was relatively large. In visible light (533–560 nm), the correlation is highly negative. After 697 nm, the correlation tends to be stable and continues to increase. Through the significance level test of p=0.01, the band with a correlation greater than 0.65, was finally extracted as the sensitive band. This significant band range was 403–475 nm, with a total of 76 bands, accounting for 43.18% of the overall variable. They are 533.3 nm, 536.7 nm, 540 nm, 543.4 nm, 546.7 nm, 550.1 nm, 553.4 nm, 556.8 nm, 560.1 nm, 697.1 nm, 700.6 nm, 704.1 nm, 707.6 nm, 711.1 nm, 767.6 nm, and 771.1–990.4 nm.

### 3.2. Selection of Characteristic Band Based on SCARS Algorithm

**Figure 7:**
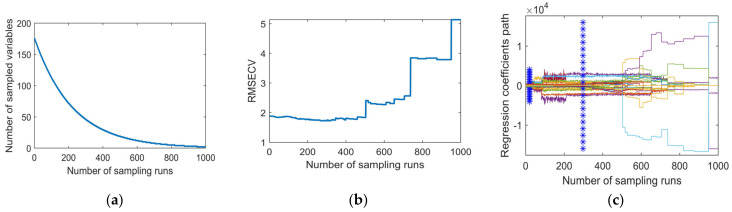
Characteristic variable selection process of sCARS algorithm. (**a**) Changes in the number of waveband variables. (**b**) Validation of RMSECV. (**c**) Path of variable regression coefficients.

Using the original spectrum as the input spectrum, the specific calculation process of the sCARS algorithm is shown in Figure 7. Figure 7a shows that as the number of sCARS iterations increases, the number of wavelengths retained gradually decreases. The decrease speed is from fast to slow, indicating that sCARS has two stages, “rough selection” and “selection” in the process of screening characteristic bands. Figure 7b shows the change in trend of 10-fold cross-validation, which has a trend from large to small and then to large. When the operation reaches 459 times, the value is the smallest, which means that at 459 times, the wavelength that affects the SPAD value modeling of the pepper leaf is eliminated. The smallest is the best selection of the band subset, and a total of 46 bands were selected, accounting for 26.14% of the overall variable. They are 386.6 nm, 392.9 nm, 402.5 nm, 415.4 nm, 431.5 nm, 526.7 nm, 530.0 nm, 590.5 nm, 593.9 nm, 597.3 nm, 600.7 nm, 610.9 nm, 614.3 nm, 617.7 nm, 624.6 nm, 641.7 nm, 645.1 nm, 676.2 nm, 679.7 nm, 683.2 nm, 693.6 nm, 711.1 nm, 718.1 nm, 732.2 nm, 832.1 nm, 850.2 nm, 853.8 nm, 868.4 nm, 872.0 nm, 875.7 nm, 879.3 nm, 890.3 nm, 894.0 nm, 916.0 nm, 919.7 nm, 923.4 nm, 927.1 nm, 930.8 nm, 938.2 nm, 945.6 nm, 953.0 nm, 960.5 nm, 971.7 nm, 979.2 nm, 982.9 nm, and 986.7 nm.

### 3.3. Selection of Characteristic Band Based on IRIV Algorithm

**Figure 8:**
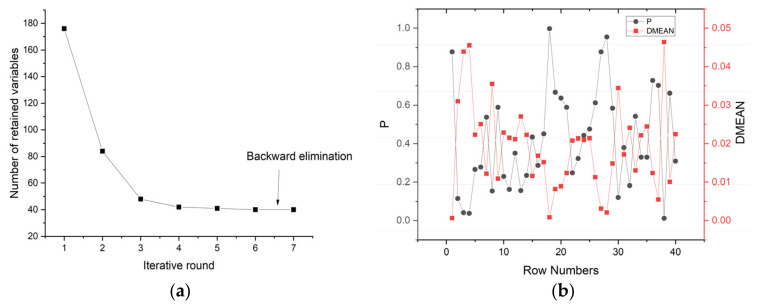
IRIV algorithm selection process: (**a**) The change in the number of retained informative variables in each round; (**b**) Changes in P value and DMEAN in the sixth round.

The purpose of the IRIV algorithm is to eliminate irrelevant variables and retain variables related to the SPAD value of pepper leaves. The algorithm uses a 5-fold cross-validation method to establish a partial least-squares model to select the characteristic variables. The IRIV algorithm has gone through seven rounds. As shown in Figure 8, the number of iteration variables in the first three rounds decreased rapidly, from 176 to 48, and then the rate of variable reduction slowed down. After the 6th iteration, the uninformative variables and interfering variables are completely eliminated. In general, only variables with a large amount of information are selected as the best set of variables. Although they have significant positive effects, they are not always optimal because the positive effects of weakly informative variables are ignored. Thus, weakly informative variables are retained at this stage. Therefore, IRIV is used to search for important variables through multiple iterative loops until there are no uninformative or interfering variables, and the optimal characteristic wavelength variable is obtained through reverse elimination. A total of 26 bands were selected, accounting for 14.77% of the overall variables. They are 477.1 nm, 490.3 nm, 510.1 nm, 526.7 nm, 597.3 nm, 600.7 nm, 610.9 nm, 614.3 nm, 617.7 nm, 624.6 nm, 628 nm, 638.3 nm, 648.6 nm, 676.2 nm, 725.1 nm, 728.7 nm, 839.3 nm, 853.8 nm, 861.1 nm, 868.4 nm, 875.7 nm, 879.3 nm, 894 nm, 916 nm, 945.6 nm, and 979.2 nm.

### 3.4. Screening Results

**Figure 9:**
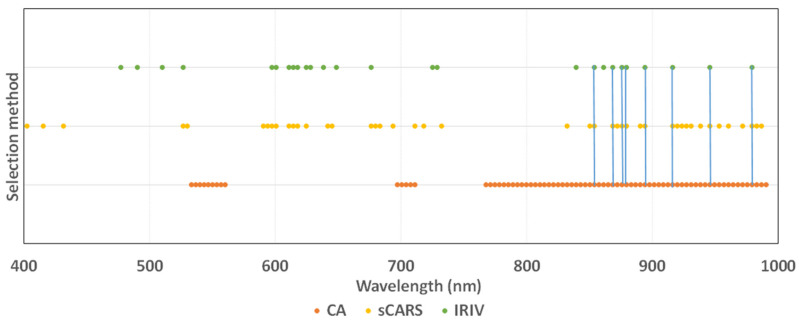
Comparison chart of optimal variable distribution.

As shown in Figure 9, the order of the three methods used to simplify the model capacity is as follows: IRIV > sCARS > CA. The CA, sCARS, and IRIV algorithms selected 76, 46, and 26 characteristic variables for modeling, accounting for only 43.18%, 26.14%, and 14.77% of the entire band, respectively. The sensitive wavelengths of pepper leaf SPAD value were concentrated between 415.4–431.5 nm, 526.7–676.2 nm, and 839.3–979.2 nm, indicating that these three bands are closely related to pepper leaf SPAD value, as shown in Figure 9, where the blue line part is the same part of the band selected by the three feature selection methods, and they are 853.8 nm, 868.4 nm, 875.7 nm, 879.3 nm, 916 nm, 945.6 nm, and 979.2 nm. This may be related to the plant nutritional status. When the nutritional status is good, the content of chlorophyll in leaves is high, there are more cell layers, and the gap between mesophyll and cells is thick, which can further increase the spectral reflectance. Finally, the higher the SPAD value, the higher the reflectance, and the same correlation is also high, which provides a reliable mathematical basis for the chlorophyll diagnosis model of pepper leaves [34].

### 3.5. Optimal Algorithm Selection

#### 3.5.1. Accuracy Comparison of Different Methods

A comprehensive comparison of the model prediction results established by different variable selection methods can be seen in Table 2. According to the 10-fold cross-validation discriminant results, the model based on the characteristic variables of the IRIV algorithm achieves the highest accuracy, and the modeling accuracy of each model is relatively high. $R_{cv}^{2}$ is above 0.8, and the accuracy of the four models constructed by it is much greater than that of the other three methods. It can be seen that the IRIV method is an effective variable selection method and is better than the full band, CA, and sCARS methods. This also shows that the IRIV algorithm is an effective means of improving the accuracy of model prediction and can improve modeling efficiency. In addition, a comparison of the four modeling methods indicates that the characteristic variable modeling of the PLSR algorithm achieves the highest accuracy. However, in terms of the overall accuracy, there is not much difference among the four models.

#### 3.5.2. Model Construction Based on the Bands selected by the IRIV Algorithm

**Figure 10:**
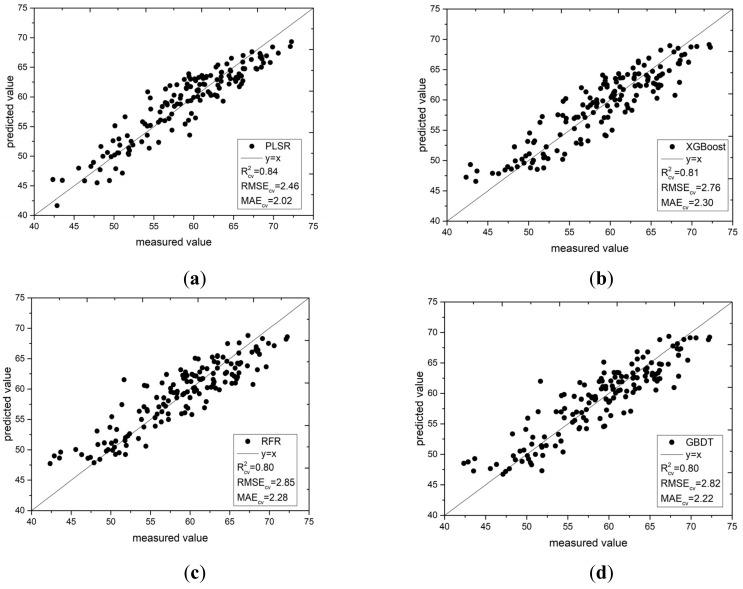
Scatter plot of measured and predicted values of the four models: (**a**) PLSR; (**b**) XGBoost; (**c**) RFR; and (**d**) GBDT.

Figure 10 shows a scatter plot of the four estimation models under IRIV feature variable screening. From the fitting effect, the results of the four modeling methods were evenly distributed on both sides of the 1:1 straight line. This shows that selecting effective feature variables from the full band spectral data and using these feature variables to build a prediction model can not only greatly simplify the model and reduce the amount of model calculations, but also improve the prediction ability and robustness of the built model. It also shows that the model constructed using this method can be used in the actual monitoring of the SPAD value of pepper leaves.

### 3.6. Chlorophyll Distribution of Pepper Leaves

Using the IRIV-XGBoost model, we estimated the SPAD value of each pixel of pepper leaves, and then drew the chlorophyll distribution map of the pepper leaves. Each SPAD value corresponds to a specific color in the color table. The specific steps are as follows:

Step 1: Hyperspectral images of pepper leaves were obtained under 26 characteristic wavelengths selected by the IRIV algorithm.

Step 2: The reflectivity of each pixel in the characteristic wavelength image was extracted.

Step 3: The SPAD value of each pixel was calculated, and a gray distribution map was obtained.

Step 4: The gray distribution map was used to draw the SPAD distribution map of the pepper leaves at different leaf positions.

**Figure 11:**
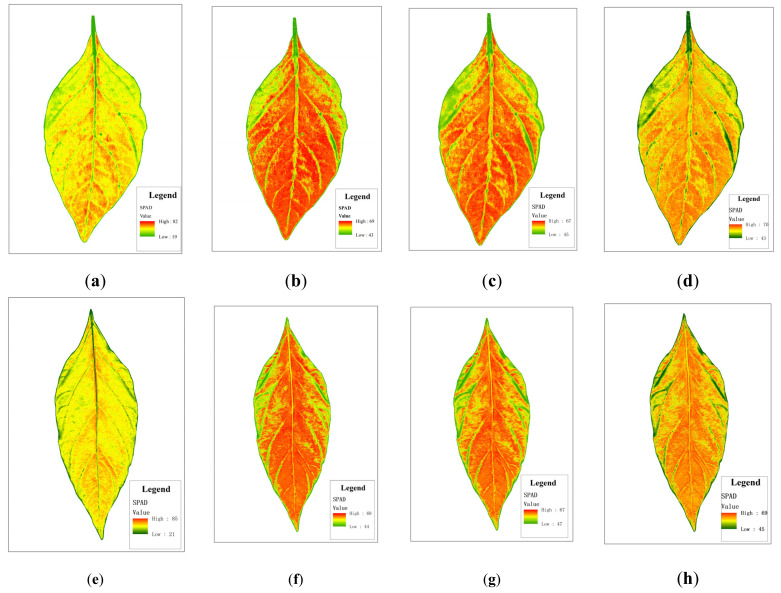
Distribution of SPAD value in the lower leaf in different models: (**a**,**e**—PLSR), (**b**,**f**—XGBoost), (**c**,**g**—RFR), (**d**,**h**—GBDT).

**Figure 12:**
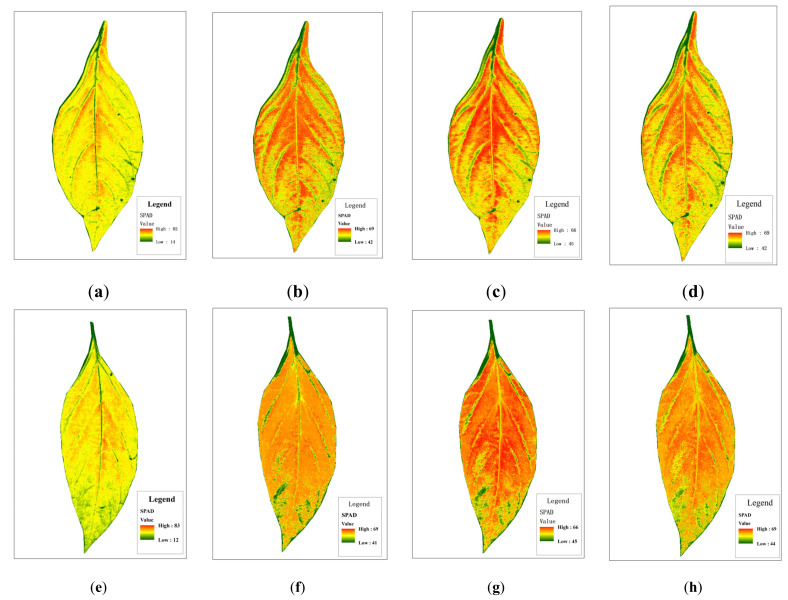
Distribution of SPAD value in the middle leaf in different models: (**a**,**e**—PLSR), (**b**,**f**—XGBoost), (**c**,**g**—RFR), (**d**,**h**—GBDT).

**Figure 13:**
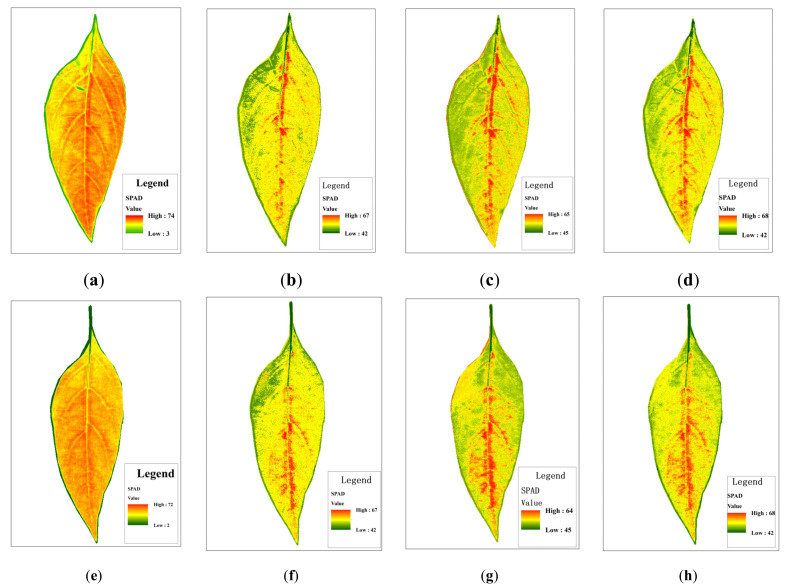
Distribution of SPAD value in the upper leaf in different models: (**a**,**e**—PLSR), (**b**,**f**—XGBoost), (**c**,**g**—RFR), (**d**,**h**—GBDT).

As shown in Figure 11, Figure 12 and Figure 13, different colors (green, yellow, and red) and color depth represent the SPAD value of pepper leaves at different concentrations. Overall, leaf chlorophyll spreads around the central vein. In the lower leaf, the overall color was evenly distributed, and the yellow and red were darker, while the middle leaf and upper leaf chlorophyll were lighter in yellow and red. The distribution of SPAD value in different leaf positions can be seen intuitively: lower > middle > upper, which is consistent with the actual measurements regarding the distribution and changes in the trend of pepper leaf SPAD values, as well as with the growth law of the pepper seedling stage.

As shown in Table 3, the statistical information of the inversion graph constructed by the three nonlinear models of XGBoost, RFR, and GBDT is relatively close to the true value, while the statistical results of the linear model PLSR show a maximum value of 82 and a minimum value of 2. This is inconsistent with the actual situation. In terms of overall performance, IRIV-XGBoost performed the best.

### 3.7. Statistical Summary Based on the IRIV-XGBoost Algorithm

**Figure 14:**
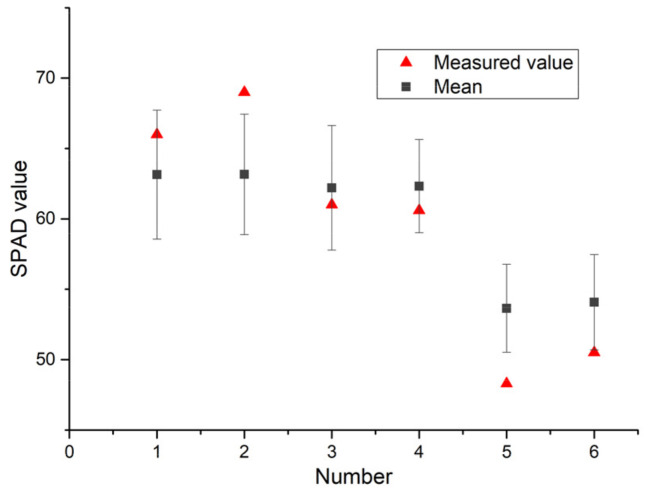
Predicted SPAD value and measured value with the standard deviation as error bars (No.1,2—Figure 11b,f, No.3,4—Figure 12b,f, No.5,6—Figure 13b,f).

The SPAD inversion images of pepper leaves obtained by the IRIV-XGBoost algorithm were separately counted. From the mean and standard deviation of each pixel of the inversion image (Figure 14), most of the predicted values are consistent with the measured values, and the predicted and measured values have strong correlation. This shows that the use of hyperspectral imaging technology to construct the SPAD distribution map of pepper leaves is effective, realizes the rapid and accurate acquisition of the SPAD of pepper leaves at a small area scale, and provides a theoretical basis for later crop growth and the development of new equipment.

## 4. Discussion

CA, sCARS, and the IRIV algorithm respectively select 76, 46, and 26 characteristic variables for modeling. The results show that prior to modeling, screening the characteristic variables of the original spectrum not only ensures the accuracy of the model, but also greatly reduces the complexity of the model. There are two reasons for this phenomenon: (1) A large number of spectral bands in hyperspectral data provides us with rich spectral information. At the same time, it also leads to redundant information and increases the complexity of data processing, which increases the calculation deviation of statistical parameters. The extraction of characteristic parameters can effectively reduce the dimension of hyperspectral data so as to achieve the effect of optimizing the model [35,36]. (2) The IRIV strategy considers the synergetic effect among variables through random combination. By means of this, only strongly informative and weakly informative variables are retained in each round. This is due to their positive effect under the condition of random combinations among variables. When compared with two outstanding variable selection methods, the outstanding performance of IRIV indicates that it is a good alternative to variable selection in multivariate calibration [22].

The three nonlinear models, XGBoost, RFR, and GBDT, obtained similar results in the hyperspectral imaging inversion stage. All three achieved good results and conformed to the measured value distribution and growth law of pepper. However, the PLSR does not match the actual situation in the inversion stage. Although the accuracy of PLSR in the modeling stage was slightly higher than that of the other three models, it performed poorly in the inversion stage. This is because the PLSR model is a linear model, and it has certain limitations when dealing with high-dimensional data. PLSR can solve the problems of multiple variables and multiple correlations between variables, but it will lose part of the effectiveness after the principal component transformation of the data. Therefore, PLSR is weak in solving nonlinear problems [37,38], and the three nonlinear models of XGBoost, RFR, and GBDT can better solve the complex nonlinear relationship between hyperspectral images and SPAD value. The model has good anti-noise ability, high model accuracy, and good robustness [39].

As shown in Figure 11, Figure 12 and Figure 13, the SPAD value of pepper leaves exhibited a stepped distribution. The farther away from the center of the plant, the lower the chlorophyll index value. The lower leaves contained higher SPAD value than the upper leaves. The reasons for this analysis may be as follows: (1) Chlorophyll is a light-absorbing substance and an important nutritional indicator. Plant nutrients are transported from the stem upward through the center of the plant to the edge of the leaf, so the SPAD value in the center of the plant is slightly higher than in the edge of the leaf. (2) Since the collected pepper plants are in the seedling stage and the lower leaves are still in the vigorous growth period, they contain more mesophyll, and the leaf functions characterized by chlorophyll are growing vigorously. The leaves are only formed during the seedling stage, and they are in a vigorous growth period. Respiration was strong. Although the stomatal conductance is high, many internal structures are imperfect, so the SPAD value is relatively low. As the leaf age increased, the leaf structure became complete, and the SPAD value gradually increased.

## 5. Conclusions

Hyperspectral data for pepper leaves located at different positions on the plant were collected to analyze the differences in the SPAD value distribution and the dynamic characteristics of the growth period of the pepper plants. The average spectra of the SPAD value measurement positions of pepper leaves were extracted, and CA, sCARS, and IRIV were used to screen feature bands. These methods were combined with PLSR, XGBoost, RFR, and GBDT to construct regression models, and the distribution of SPAD value in pepper leaves at different leaf positions was drawn. The main conclusions of this study are as follows:

(1) A comprehensive comparison of the full band, CA, sCARS, and IRIV variable screening feature bands was undertaken to construct a variety of SPAD value estimation models and the model capabilities were tested through 10-fold cross-validation. The estimation capabilities of the different models were quite different. The IRIV algorithm achieved the highest accuracy, above 0.8, which greatly reduces the complexity of the model while ensuring the accuracy of the model.

(2) Four modeling methods were compared: PLSR, XGBoost, RFR, and GBDT. The accuracy of PLSR in the modeling stage is slightly higher than that of the other three models, but it performs poorly in the inversion stage. XGBoost is better suited to solve the complex nonlinear relationship between hyperspectral images and SPAD value. The model has good anti-noise ability, high model accuracy, and good robustness.

(3) The IRIV-XGBoost model was used to calculate the SPAD value of each pixel of pepper leaves and then invert the chlorophyll distribution map of pepper leaves at different leaf positions, which can reflect the dynamic response of pepper leaf chlorophyll in plants in the seedling stage and finally realize the non-destructive detection of pepper leaf content for different leaves and the visual expression of chlorophyll distribution. This result is consistent with the distribution and change trend of the SPAD value of pepper leaves when measured, and it is also in line with the growth law of pepper seedling stage. In future, the dynamics of different growth periods need to be tested and verified in the field to lay a foundation for the overall dynamic diagnosis of pepper canopy.

## Figures and Tables

**Table 1:** Variable classification rules.

Wavelength Variable Type	Classification Rules
Strongly informative	$DMEAN_{i}<0$ $,\;P_{i}<0.05$
Weakly informative	$DMEAN_{i}<0$ $,\;P_{i}>0.05$
Uninformative	$DMEAN_{i}>0$ $,\;P_{i}>0.05$
Interfering	$DMEAN_{i}>0$ $,\;P_{i}<0.05$

**Table 2:** Comparison of accuracy of different methods.

**Selection Method**	**Number of Bands**	**Modeling Algorithm**	$R_{cv}^{2}$	$RMSE_{cv}$	$MAE_{cv}$
Full bands	176	PLSR	0.52	2.57	2.11
		XGBoost	0.48	2.80	2.28
		RFR	0.42	2.95	2.83
		GBDT	0.50	2.76	2.19
CA	76	PLSR	0.48	2.59	2.1
		XGBoost	0.29	3.00	2.39
		RFR	0.41	2.95	2.4
		GBDT	0.44	2.84	2.23
sCARS	46	PLSR	0.55	2.59	2.13
		XGBoost	0.54	2.68	2.17
		RFR	0.43	2.92	2.32
		GBDT	0.53	2.74	2.17
IRIV	26	PLSR	0.84	2.46	2.02
		XGBoost	0.81	2.76	2.30
		RFR	0.80	2.85	2.28
		GBDT	0.80	2.82	2.22

**Table 3:** Statistical information of chlorophyll inversion map of pepper leaves under different models and different leaf positions.

Leaf Position	Measured Value	Model Method	Min Value	Max Value
Lower leaf	66.0	PLSR	19	82
		XGBoost	43	69
		RFR	46	67
		GBDT	43	70
	69.0	PLSR	21	85
		XGBoost	44	69
		RFR	47	67
		GBDT	45	69
Middle leaf	61.0	PLSR	14	82
		XGBoost	42	69
		RFR	45	66
		GBDT	42	69
	60.6	PLSR	12	83
		XGBoost	41	69
		RFR	45	66
		GBDT	44	69
Upper leaf	48.3	PLSR	3	73
		XGBoost	42	67
		RFR	45	65
		GBDT	42	68
	50.5	PLSR	2	72
		XGBoost	42	67
		RFR	45	64
		GBDT	42	68

## Data Availability

Not applicable.
